# Domperidone, Parkinson disease and sudden cardiac death: Mice and men show the way

**DOI:** 10.6061/clinics/2016(02)01

**Published:** 2016-02

**Authors:** Fulvio A Scorza, Carla A Scorza, Henrique B Ferraz

**Affiliations:** I Universidade Federal de São Paulo (EPM/UNIFESP), Escola Paulista de Medicina, Disciplina de Neurociência, São Paulo/SP, Brazil; II Universidade Federal de São Paulo (EPM/UNIFESP), Escola Paulista de Medicina, Disciplina de Neurologia, São Paulo/SP, Brazil

Sudden cardiac death (SCD) is a highly visible tragedy that generates intense debate among medical experts, members of scientific communities and laypersons alike. By definition, SCD is usually reported as unexpected death within one hour of the onset of a change in clinical status as the result of cardiovascular events in a person with or without preexisting heart disease 1,2. The pathophysiology of SCD is heterogeneous, but SCD is caused by electric instability and lethal ventricular arrhythmias followed by hemodynamic collapse 1. From an epidemiological perspective, according to recent, well-designed prospective studies conducted in different countries, SCD rates range from 50 to 100 cases per 100,000 in the general population 1,. In the same studies, “sudden” death also occurred in many patients with acute catastrophic neurological diseases or chronic neurological disorders with acute decompensation 9. Following this line of reasoning and because Parkinson disease (PD) has been neglected in this field of research, it is appropriate to consider the possible occurrence of SCD among individuals with PD and highlight these possibilities in the current scientific scenario.

PD is one of the most common, age-related neurodegenerative disorders and is characterized by tremors, muscular rigidity, slowed movement and postural imbalance that results from progressive neuronal loss in specific brain regions 10,11,12. PD affects approximately 0.3% of adult individuals in general, more than 1% of people over 60 years of age and 4% of individuals over 80 years of age 13,14. Moreover, the annual incidence for PD ranges from 8 to 18 cases per 100,000 person years 13,14. Interestingly, whether PD increases mortality remains a moot point 15. Although some studies suggest that mortality over time among PD individuals is inconsistent 15, observational, meta-analysis and systematic review studies conducted over the last decades have demonstrated that PD is a condition that, in certain situations, is accompanied by high rates of premature death compared with the general population 10,. Neurologists have attempted to identify the risk factors for sudden death in individuals with PD, but the knowledge in this area is still limited. Some of the documented risk factors of mortality include aspiration pneumonia, dementia, old age, late age of onset and male gender 10,. At the same time, a substantial proportion of individuals with PD die prematurely and suddenly. In a study developed by Rajput and Rozdilsky, one in six subjects with PD died suddenly without an identifiable toxicological or anatomical cause of death according to postmortem autopsy analyses 25,26. These researchers also conducted an epidemiological study of PD over a 13-year period (1967 through 1979) and updated preliminary reports on the incidence and trends of PD among a population in Rochester, Minnesota 27. Clearly, the mortality rate among PD patients was significantly higher than that among control subjects and was unchanged from previous rates that were described from the same community 27. In 2006, Sato et al. studied the long-term outcomes in a large cohort of Japanese people with PD (total of 1,768 subjects) who visited their clinic for more than a decade. In this report, 10 of 131 PD individuals died of sudden death 26,28,29. A recent, interesting study by Matsumoto et al. reviewed the clinical data and causes of death among 16 persons with PD who underwent postmortem examinations. In this study, a considerable amount of PD individuals died of sudden death (4 of 16), and no satisfactory causes of death were identified, even after performing an autopsy. Thus, a large number of people with PD die of sudden death 26.

Sudden death in patients with PD does not result from a specific cause. A fairly common systemic condition that accompanies PD is cardiac autonomic imbalance, which is the major mechanismrelated to sudden death in patients with PD 30,31. Despite recent increases in understanding SCD among the general population during recent decades, the dearth of published data regarding the general risk factors of sudden death occurrence in PD patients encouraged us to address this specific topic. In particular, we focused on the potential role of domperidone as a trigger agent of fatal cardiac events in individuals with PD.

Domperidone is an oral, dopamine receptor blocker that is utilized for nausea and vomiting 32,33,34. Many PD patients develop these gastrointestinal symptoms and use an anti-dopaminergic agent for treatment 35. Recent evidence indicates that domperidone has limited gastrointestinal benefits and may confer a high risk of SCD 32,36. According to several clinical studies, intravenous domperidone is associated with the occurrence of arrhythmias, QT interval prolongation, Torsades de Pointes, and ventricular fibrillation. Thus, SCD occurs when domperidone is given in doses adequate to protect against emesis in people receiving chemotherapy treatment 32,. Surprisingly, a thorough analysis of five large, population-based studies has shown an increased odds ratio for SCD in people treated with oral domperidone 32,36. Following this line of reasoning, few studies have investigated the relationship between domperidone and SCD in PD patients 35. In the early 2000s, the cardiovascular effects of domperidone in individuals with idiopathic PD were treated with continuous subcutaneous infusion of apomorphine 35,44. During treatment, the blood pressures and heart rates of 10 patients were monitored for 24 h before and after treatment with domperidone using an automatic device for blood pressure recording 35,44. Domperidone increased blood pressure and heart rate without inducing nocturnal hypertension in apomorphine-treated patients with idiopathic PD 35,44. Despite this study, the scientific knowledge regarding this subject remains limited. The elegant article by Lertxundi et al. 35 reviewed the available data and clearly demonstrated a lack of published studies regarding the serious ventricular arrhythmias or SCDs associated with domperidone intake in PD patients 35. Despite this finding, the authors also indicated that domperidone is currently available as a prescription medication in more than 50 countries and as a non-prescription medication in various countries in Europe, Asia and Latin America 35 (including Brazil). Domperidone is not authorized for use in the United States 35. We must be vigilant concerning the suggestions of the authors 35, who comment that although domperidone is still the first option for treating gastrointestinal symptoms in patients with PD, doses that exceed 30 mg/day should be used with caution because the related cardiotoxicity may trigger a fatal cardiac event 35.

Unfortunately, it is difficult to estimate the occurrence of SCD among PD patients. Current research should instead focus on identifying new risk factors (including domperidone use) and the putative biologic mechanisms, as well as on developing potential preventive measures that could be used to decrease the incidence of sudden death among patients with PD. Thus, clinicians and researchers should consider short-, medium- and long-term goals to achieve these expectations. Clinicians in various medical specialties and scientists (i.e., neurologists, cardiologists, neuroscientists, geneticists and molecular biologist) should collaborate to establish experimental and clinical protocols with more efficacy to reduce the numbers of sudden deaths among patients with PD. Clinicians should also identify new approaches that offer the possibility of prevention in the near future. Following this reasoning and considering the same proposals for people with epilepsy 45,46, it is important to consider that PD patients who are at risk require a thorough cardiovascular medical history investigation, long-term ECG recordings and cardiac MR imaging. Because the causes of SCD among PD patients remain unknown, animal models of PD have been widely used during the past four decades to investigate the pathogenesis and pathophysiology of this neurodegenerative disorder 47. Despite the wide variety of existing models, indications to utilize one PD model will depend upon the specific hypotheses of the study 47. Specifically, the animal model could potentially elucidate the common putative autonomic factors that may lead to SCD in patients with PD 48. With these considerations, translational research 49, the process of streamlining basic science findings to clinical research and then into practice for the patients who are supposed to benefit from the research (bench-to-bedside) 49, is needed to address the phenomenon of SCD in PD.

In conclusion, we still do not precisely understand the main causes and mechanisms of SCD in individuals with PD, regardless of their use of domperidone. Ultimately, prevention is still better than a cure.
